# Vdelta1 T cells are more resistant than Vdelta2 T cells to the immunosuppressive properties of galectin-3

**DOI:** 10.3389/fimmu.2023.1286097

**Published:** 2024-01-08

**Authors:** Jan Schadeck, Hans-Heinrich Oberg, Matthias Peipp, Nina Hedemann, Wolfgang W. Schamel, Dirk Bauerschlag, Daniela Wesch

**Affiliations:** ^1^ Institute of Immunology, University Medical Center Schleswig-Holstein, Christian-Albrechts University, Kiel, Germany; ^2^ Divison of Antibody-Based Immunotherapy, University Medical Center Schleswig-Holstein, Christian-Albrechts University, Kiel, Germany; ^3^ Department of Gynecology and Obstetrics, University Medical Center Schleswig-Holstein, Kiel, Germany; ^4^ Signalling Research Centre Biological Signalling Studies (BIOSS) and Centre of Integrative Biological Signalling Studies (CIBSS), Faculty of Biology, University of Freiburg, Freiburg, Germany; ^5^ Centre for Chronic Immunodeficiency (CCI), Medical Centre Freiburg, and Faculty of Medicine, University of Freiburg, Freiburg, Germany

**Keywords:** gammadelta T cells, Vdelta1, galectin-3, tumor-infiltrating T cells, ovarian cancer, integrins, TIGIT, PD-1

## Abstract

Ovarian carcinomas have the highest lethality amongst gynecological tumors. A problem after primary resection is the recurrence of epithelial ovarian carcinomas which is often associated with chemotherapy resistance. To improve the clinical outcome, it is of high interest to consider alternative therapy strategies. Due to their pronounced plasticity, γδ T cells are attractive for T-cell-based immunotherapy. However, tumors might escape by the release of lectin galectin-3, which impairs γδ T-cell function. Hence, we tested the effect of galectin-3 on the different γδ T-cell subsets. After coculture between ovarian tumor cells and Vδ1 or Vδ2 T cells enhanced levels of galectin-3 were released. This protein did not affect the cytotoxicity of both γδ T-cell subsets, but differentially influenced the proliferation of the two γδ T-cell subsets. While increased galectin-3 levels and recombinant galectin-3 inhibited the proliferation of Vδ2 T cells, Vδ1 T cells were unaffected. In contrast to Vδ1 T cells, the Vδ2 T cells strongly upregulated the galectin-3 binding partner α3β1-integrin after their activation correlating with the immunosuppressive properties of galectin-3. In addition, galectin-3 reduced the effector memory compartment of zoledronate-activated Vδ2 T cells. Therefore, our data suggest that an activation of Vδ1 T-cell proliferation as part of a T-cell-based immunotherapy can be of advantage.

## 1 Introduction

Human γδ T cells can be classified in at least three major subsets, defined by the variable domain of the δ chain (1). Alternatively, they can be classified by the variable domain of the γ chain in six major subsets (2, 3). Vδ1 T cells with a variable γ chain are present in the skin and mucosa, while Vδ3 T cells with a variable γ chain constitute a main population in skin and liver (4). Their antigens are so far not defined in detail (5, 6). Vδ2 T cells which coexpress Vγ9 are mainly found in the peripheral blood. Vγ9Vδ2 T cells recognize with their canonical T-cell receptor prenyl pyrophosphates that are enhanced in many tumor cells due to a dysregulated mevalonate pathway and are recruited into the tumor via chemokine receptors (7–9). All three human γδ T-cell subsets infiltrate in tumors including colorectal cancer, Merkel cell carcinoma and ovarian cancer and have been implicated in cancer immunosurveillance (3, 10, 11). A prognostic significance of γδ T cells in a broad range of human tumor entities, a correlation with patient outcome together with their high plasticity and their HLA-independent recognition of antigens offers interesting perspectives for γδ T-cell-based immunotherapy (12–14). Otherwise, the high functional plasticity of γδ T cells can promote γδ T-cell differentiation into an immunosuppressive phenotype (15–17).

Our recently published data revealed that the activation of Vγ9Vδ2 T cells cocultured with pancreatic ductal adenocarcinoma (PDAC) cells induced an enhanced release of the lectin galectin-3 by PDAC cells (18). Galectin-3 binds to β-galactoside and thus to glycosylated Natural killer (NK) and T-cell surface receptors thereby inducing impairment of NK cell activity and anergy of tumor infiltrating CD8 lymphocytes (CD8 TIL) in cancer (19–22). Therefore, galectin-3 is regarded as an intrinsic tumor escape mechanism (17, 23). Our previous results demonstrated that galectin-3 did not influence Vγ9Vδ2 T-cell cytotoxicity against PDAC cells but inhibited their proliferation by interacting with the glycosylated receptor α3β1 integrin (CD49c/CD29) on the cell surface, which is of high relevance for γδ T-cell-based immunotherapy (18).

Here, we were interested whether other tumor entities such as epithelial ovarian tumors (EOC) cocultured with Vγ9Vδ2 T cells release comparable amounts of galectin-3 as the cross talk of PDAC and Vγ9Vδ2 T cells did. Recently, others described a negative correlation with the overall survival rate, a platinum resistance and a correlation with pathologic grading in EOC patients which highly express galectin-3 and p65 (24, 25). As shown in experimental animal tumor models, targeting the interaction of galectin-3 with N-glycosylated ectodomain MUC16 expressed on serous ovarian cancer cells by high-affinity antibody is suggested as a potential cancer therapeutic strategy (26, 27).

More interestingly, the effector function of Vγ9Vδ2 T cells was examined after cross talk with ovarian cancer cells in comparison to Vδ1 T cells, since the number of tumor infiltrating Vδ1 T cells (Vδ1 TIL) is increased within pancreatic and ovarian tumor tissue. A different sensitivity of both γδ T-cell subsets towards immunosuppressive properties of galectin-3 could have consequences for γδ T-cell-based immunotherapy.

## 2 Materials and methods

### 2.1 Patient cohort

Leukocyte concentrates from healthy adult blood donors were provided by the Department of Transfusion Medicine of the University Medical Center Schleswig-Holstein (UKSH) in Kiel, Germany. EDTA blood and tumor tissue from patients were obtained from the Department of Gynecology and Obstetrics of the UKSH in Kiel, Germany. Written informed consent was obtained from all donors in accordance with the Declaration of Helsinki, and the research was approved by the relevant institutional review boards (Ethic Committee of the Medical Faculty of the CAU Kiel, code number: D 445/18).

### 2.2 Established and freshly isolated tumor cell lines and their culture conditions

The human PDAC cell line PancTuI was kindly provided by Dr. C. Röder and Prof. Dr. A. Trauzold, Institute for Experimental Cancer Research, Kiel, Germany. Esophageal adenocarcinoma OE-33 cell line, UM-UC-3 bladder cancer cell line, non-small cell lung adenocarcinoma NCI-H1693 cells, breast cancer cells (MCF-7, MDA-MB-231) and ovarian cancer cells (OVCAR-3, BG-1 and SKOV-3) were ordered from ATCC, Manassas, VA, USA. Freshly isolated KIEL-Ovarian Cancer primary (KI-OCp) cells derived from tumor tissue (Gynecology Department, UKSH, Kiel) were prepared as described elsewhere (3). Briefly, tumor tissues were minced and treated with components A, H, and R of the Tumor Dissociation Kit (Miltenyi Biotec, Bergisch Gladbach, Germany) for 1 h at 37°C in 5 mL PBS in a gentle MACS (Miltenyi Biotec). Whereas KI-OCp1, 012 and 15 were passaged over several times, freshly isolated ovarian tumor cells KI-OCp79, 88 and 91 were used directly after tumor dissociation. All tumor cells were cultured in complete medium under regular conditions (5% CO_2_, humidified, 37°C). For dissociation of the adherent tumor cell lines from flasks, 0.05% trypsin/0.02% EDTA (Sigma Aldrich/Merck, Darmstadt, Germany) was used. The cells were then collected, centrifuged and individual amounts of tumor cells were disseminated with medium in flasks again. Absence of mycoplasma was routinely confirmed by RT-PCR (Venor R GEM classic, Minerva Biolabs GmbH, Germany) and genotype by short tandem repeat analysis.

### 2.3 Isolation of peripheral blood mononuclear cells and tumor-infiltrating lymphocytes

Peripheral blood mononuclear cells (PBMC) were isolated from the leukocyte concentrates or from EDTA blood of ovarian patients by Ficoll-Hypaque™ PLUS (Cytiva, Uppsala, Sweden) density gradient centrifugation. Cells were washed in PBS, and resuspended in RPMI 1640 (Gibco, Paisley, Scotland) supplemented with 2 mM L-glutamine, 25 mM Hepes, 100 U/mL penicillin, 100 μg/mL streptomycin (PanReac AppliChem, Darmstadt, Germany), 10% FCS (Thermo Fisher Scientific, Langenselbold, Germany) (complete medium). Tumor-infiltrating cells (TIL) were isolated from the dissociated tumor tissue described under 2.2. Digested cell suspension was passed after the gentle MACS through a 100 µm cell strainer (Falcon, BD Biosciences, Heidelberg, Germany), and centrifuged at 481 ×g for 5 min. TIL and tumor cells were separated by Ficoll-Hypaque (Biochrom, Berlin, Germany) density gradient centrifugation followed by an adherence step for several hours.

The percentage of Vδ1 T cells within PBMC ranged between 0.1 and 3% (median 0.5%), and in TIL between 0.5 and 5% (median 1%), whereas the percentage of Vδ2 T cells within PBMC ranged between 0.1 and 10% (median 1.7%), and in TIL between 0.1 and 2.5% (median 0.9%).

### 2.4 Establishment of γδ T-cell lines out of PBMC or TIL

Το expand Vγ9Vδ2-expressing T cells, 1x10^6^ PBMC or TIL/mL were stimulated with 2.5 μM aminobisphosphonate (n-BP) zoledronate (Novartis, Basel, Switzerland), which induces selective activation and proliferation. The expansion of Vδ1-expressing γδ T cells was induced by coating 24-well plates with 250 μL/well of 10 μg/mL anti-Vδ1 mAb clone R9.12 (Beckmann Coulter, Krefeld, Germany) overnight at 4°C and washing the wells afterwards. 1x10^6^ PBMC or TIL/well were cultured in the coated wells with soluble 1 μg/mL anti-CD28 mAb clone CD28.2 (BioLegend, San Diego, USA). In one patient Vγ2,3,4-expressing Vδ1 T cells were expanded by incubating PBMC with anti-Vγ2,3,4 mAb clone 23D12 (28) and cross-linking via rabbit-anti-mouse polyclonal Ab (Dianova, Hamburg, Germany) for a period of 30 minutes each. After washing, 1x10^6^ PBMC/well were cultured with 50 IU/mL rIL-2 and 1 μg/mL anti-CD28 mAb clone CD28.2 for a period of 14 days. Since resting, initially stimulated γδ T cells produced only low amounts of IL-2, 50 IU/mL of recombinant IL-2 was added every 2 days over a culture period of 14 days.

After 14 days, the short-term activated γδ Τ-cell lines were stained with AF700-labeled anti-CD3 clone SK7 (BioLegend), AF488-labeled anti-Vγ9 clone 7A5 (29), AF647-labelled anti-Vγ2,3,4 clone 23D12 (28) PE-Cy7-labeled anti-T-Cell Receptor (TCR) pan γδ clone 11F2, PE-labeled anti-Vδ2 clone B6 (both BD Biosciences, Heidelberg, Germany) and with VioBlue-labeled anti-Vδ1 clone REA173 (Miltenyi Biotec, Bergisch Gladbach, Germany), and analyzed by flow cytometry to determine the purity. At a purity of >95%, γδ T cells were used for functional assays or preserved in liquid nitrogen, while they were subjected to a positive magnetic separation by using anti-Vγ2,3,4 mAb clone 23D12 or anti-Vδ1 mAb clone R9.12 followed by anti-Mouse IgG MicroBeads (Miltenyi Biotec) at a purity <95%. After positive selection, cells were restimulated in rIL-2-supplemented medium with 0.5 μg/mL phytohaemagglutinin (Thermo Fisher Scientific), and irradiated PBMC (20x10^6^ cells) and/or EBV-transformed B cell lines (2x10^6^ cells) as feeder cells for 20x10^6^ γδ T cells. Dead feeder cells were removed 3-4 days after restimulation by Ficoll-Hypaque density gradients. Purity of γδ T cells was >95% as analyzed by flow cytometry.

### 2.5 Functional cell culture assay

To analyze the effect of galectin-3 (BioLegend) on the proliferation of γδ T cells, the percentage of γδ T cells was determined and accordingly adapted to 2x10^4^ γδ T cells per well. Therefore, the number of PBMC and TIL ranged between 1.5 to 7x10^5^ PBMC or TIL per 24-well. PBMC or TIL were plated in complete medium with 50 IU/mL rIL-2. These cells were selectively activated by either 2.5 μM zoledronate or by coating the wells with 250 μL/well of 10 μg/mL anti-Vδ1 mAb clone R9.12 together with soluble 1 μg/mL anti-CD28 mAb clone CD28.2 in the absence or presence of different concentrations (0.01, 0.1, 1, 10, 1000 ng/mL) of galectin-3 or 100 nM galectin-3 inhibitor TD-139 (Selleck Chemicals, Planegg, Germany) daily. After 6, 7 and/or 9 days, the Vδ1 and Vδ2 T-cell proliferation was analyzed as described in the ‘absolute cell number analysis by Flow Cytometry’ section.

When coculturing tumor cells to determine absolute cell numbers or perform galectin-3 ELISA, 20x10^3^ PDAC cells (PancTuI) or different ovarian cancer cells were plated in 24-well plates in complete medium. After 24 hours, a calculated amount of PBMC were added to reach an effector/target (E/T) ratio of 1:1 (Vδ1 or Vδ2 T cells/tumor cells) PBMC were either coculture in 50 IU/mL rIL-2 in complete medium only, with 2.5 μM zoledronate or with bispecific T-Cell Engagers (bsTCE) selectively targeting human epidermal growth factor receptor (HER)-2 expressing ovarian tumor cells to Vγ9Vδ2 (30) or Vδ1 T cells (Oberg et al., manuscript in preparation).

To determine the expression of differentiation (naïve, central and effector memory, TEMRA), activation (CD25, CD69) and immune check point markers (TIGIT, PD-1) or galectin-3 binding partner α3β1 (CD49c/CD29), 5x10^5^ PBMC were cultured in complete medium, and stimulated with 2.5 μM zoledronate or with coated anti-Vδ1 mAb clone R9.12 (10 μg/mL) together with soluble anti-CD28 mAb clone CD28.2 (1 μg/mL).

For CD49c/CD29 expression, 5x10^5^ PBMC were additionally cocultured with 5x10^4^ OVCAR-3 cells in the presence of bsTCE selectively targeting HER-2 expressing ovarian tumor cells to Vγ9Vδ2 or Vδ1 T cells.

Cells were stained as described in flow cytometry section after time points indicated in the figures.

### 2.6 Flow cytometry

In total, 1x10^6^ PBMC from healthy donors or cancer patients, TIL from ovarian cancer patients and generated γδ T-cell lines were stained by multicolor flow cytometry approach to distinguish between diverse γδ T-cell subsets within different CD45^+^ leukocyte populations for usage in functional assays. The color panel included the following backbone mAb: PerCP-labeled anti-CD45 clone 2D1, PE-Cy7-labeled anti-TCR pan γδ clone 11F2 (both BD Biosciences), AF700-labeled anti-CD3 clone SK7, BV510-labeled anti-CD4 clone OKT4 (both BioLegend), and additional mAbs: VioBlue-labeled anti-Vδ1 clone REA173 (Miltenyi Biotec), PE-labeled anti-Vδ2 clone B6 (BD Biosciences), AF488-labeled anti-Vγ9 clone 7A5 (29), AF647-labelled anti-Vγ2,3,4 clone 23D12 (28), PEeFluor610-labeled anti-CD56 clone CMSSB (Thermo Fisher Scientific), APC-Cy7-labeled anti-CD8 clone SK1 and BV605-labeled anti-CD19 clone HIB19 (both Biolegend).

To determine activation, immune check point and differentiation marker, we combined backbone mAbs with PE-labeled anti-CD25 mAb clone REA945 (Miltenyi Biotec), APC-labeled anti-CD69 mAb clone FN50 (Biolegend), BV711-labeled anti-TIGIT mAb clone 741182 (BD Biosciences), BV785-labeled anti-PD-1 (CD279) mAb clone EH12.2H7 (Biolegend), BV605-labeled anti-CD45RA mAb clone HI100, PE-Dazzle594-labeled anti-CD27 mAb clone O323 (both Biolegend), APC-Vio770-labeled anti-Vδ2 mAb clone REA771 and VioBlue-labeled anti-Vδ1 clone REA173 (Miltenyi Biotec).

To analyze expression of CD49c and CD29, cells were stained with the backbone mAb together with APC-Vio770-labeled anti-Vδ2 mAb clone REA771, VioBlue-labeled anti-Vδ1 mAb clone REA173, PE-labeled anti-CD49c mAb clone C3 II.1 (BD Biosciences) and AF647-labeled anti-CD29 mAb clone TS2/16 (Biolegend).

Alternatively, a color panel described in the absolute cell number section was used for staining the PBMC, TIL and γδ T-cell lines at d0, d6, d7 and d9 of the functional assays.

To determine the expression of tumor-associated antigens such as epithelial cell adhesion molecule (EpCAM) and HER-2, 1x10^5^ of tumor cells were stained with the following mAbs: PerCP-labeled anti-CD45 clone 2D1 (BD Biosciences), PE-Vio770-labeled anti-HER-2 clone 24D2 and APC-labeled anti-EpCAM clone HEA-125 (both from Miltenyi Biotec) and corresponding isotype controls (BD Biosciences or Miltenyi Biotec). All tumor cells were also intracellularly stained with AF647-conjugated anti-galectin-3 mAb clone M3/38 or AF647-conjugated isotype rat IgG2a mAb clone RTK2758 (both BioLegend). For the intracellular staining, 2-5x10^5^ cells were washed with staining buffer, fixed and permeabilized with Cytofix/Cytoperm kit (BD Biosciences), for 20 min following the procedures outlined by the manufacturer. Thereafter, the cells were washed twice with Perm/Wash by centrifugation and stained with anti-galectin-3 or isotype control mAb for 25 min, washed again twice and measured. All samples were analyzed on LSR-Fortessa flow cytometer (BD Biosciences) using Diva 9 software.

### 2.7 Absolute cell number analysis by flow cytometry

After culturing PBMC or TIL in the presence or absence of tumor cells, Trucount Tubes (#340334 from BD Biosciences) were used to measure the absolute cell number of viable Vδ1, Vδ2 and tumor cells. Therefore, supernatant was collected from the wells to determine galectin-3 release, and the remaining cells were transferred from the wells into 1.5 mL reaction tubes. To dissociate and collect the adherent cells, 0.05% trypsin/0.02% EDTA was used. After a washing step, the cells were stained with PerCP-labeled anti-CD45 mAb clone 2D1, PE-Cy7-labeled anti-TCR pan γδ mAb clone 11F2 (both BD Biosciences), AF700-labeled anti-CD3 mAb clone SK7 (BioLegend), APC-Vio770-labeled anti-Vδ2 mAb clone REA771, VioBlue-labeled anti-Vδ1 mAb clone REA173, APC-labeled anti-EpCAM mAb clone HEA-125 (all three Miltenyi Biotec) and then washed again. For the differentiation between viable and dead cells, SYTOX™ Green dead cell stain with a dilution of 1:4000 (Thermo Fischer Scientific, order number S34860) was added to the probes with washing buffer. These were transferred into the Trucount tubes and analyzed on LSR-Fortessa flow cytometer (BD Biosciences) using Diva 9 software. Because each Trucount Tube contains a defined amount of beads the absolute cell number of the different cell populations can be calculated by dividing the total amount of beads by the measured beads with the flow cytometer and using this digit to multiply the various cell populations (**Supplementary Figure 1**).

### 2.8 Enzyme-linked immunosorbent assay

The quantification of galectin-3 released by tumor cells, PBMC and TIL alone or after coculture of these subsets was measured by sandwich DuoSet ELISA kit (#DY1154 from R&D System, Wiesbaden, Germany) in duplicates following the procedures outlined by the manufacturer. For this measurement 500 μL of the supernatants were collected after different incubation times (24 hours to 9 days), centrifuged and 2 x 200 μL were stored at -20°C until use.

### 2.9 Real-time cell analyzer

The cytotoxicity of the γδ T-cell lines against adherent cancer cell lines was analyzed in triplicates by using an RTCA (x-Celligence, Agilent Technologies, Inc., Santa Clara, CA, USA). The tumor cells were added to a 96-well micro-E-plate (10.000 cells per well) with complete medium to monitor the impedance via electronic sensors every 5 minutes for up to 24 hours, which can be equated with the adherence of the tumor cells. After the tumor cells have reached a linear growth phase, γδ T-cell lines with 50 IU/mL rIL-2 were added to the 96-well micro-E-plate. The cells were cultured in medium or stimulated with bsTCE such as [(HER2)_2_×Vγ9] and [HER2×CD3] described elsewhere (30) or an unpublished bsTCE targeting HER2-expressing ovarian tumor cells to Vδ1 T cells (manuscript in preparation). In addition, galectin-3 or titrated galectin-3 inhibitor TD-139 (Selleck Chemicals, Planegg, Germany) were added. Impedance variations are shown in an arbitrary unit called cell index (CI) which correlates with adherence and spreading, cell proliferation and cell death (in this case the tumor cells). To compare the ability of the different γδ T-cell lines to lyse the tumor cells and due to slight differentiation before adding the substances and T cells, the CI was normalized to 1. Triton X-100 with the final concentration of 1% per well was added to 3 wells with tumor cells only as a positive control for tumor cell death. The mean of Triton-X-100 samples was calculated and defined as 100% lysis after 4, 12 and 24 hours. The raw data files were exported from the RTCA software 2.0 to Microsoft Excel to calculate the cytotoxic potential of the γδ T-cell lines towards tumor cells. The ratio of each sample to spontaneous lysis of tumor cells alone was calculated and the ratio was normalized to maximal inducible lysis by Triton-X-100.

### 2.10 Statistical analysis

GraphPad Prism (GraphPad Software, LLC., La Jolla, CA, USA) was used for statistical analysis. The Shapiro-Wilk test was applied to determine the normal distribution assumption. For parametric data of matched datasets, a paired, two-tailed t-test was used. For non-parametric data of matched datasets, a Wilcoxon matched-pairs signed rank test was used. All statistical tests were two-sided, and the level of significance was set at α ≤ 5%. The appropriate tests are indicated in the figure legends.

## 3 Results

### 3.1 Cross talk of tumor cells with activated Vδ2 T cells induces enhanced release of galectin-3

**Figure 1:**
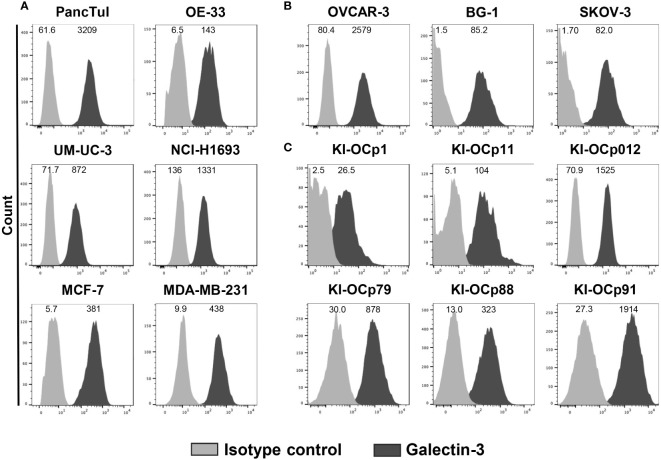
Intracellular galectin-3 expression in different tumor cells. **(A–C)** Median fluorescence intensity (MFI) (n = 13) of galectin-3 expression (clone M3/38) and isotype control is shown for the indicated cell lines measured by LSR-Fortessa. The grey histograms represent the isotype control and the black histograms the galectin-3 expression.

Recently, we demonstrated that galectin-3 produced by PDAC cells inhibited Vγ9Vδ2 T-cell proliferation (18). Therefore, we asked whether the expression and release of galectin-3 is a common tumor escape mechanism of different tumor cells. Comparable to PDAC cells (PancTuI), esophageal adenocarcinoma OE-33 cell line, the UM-UC-3 bladder cancer cell line, non-small cell lung adenocarcinoma NCI-H1693 cells, breast cancer cells (MCF-7, MDA-MB-231) and ovarian cancer cells (OVCAR-3, BG-1 and SKOV-3) expressed intracellular galectin-3 (**Figures 1A, B**).

OVCAR-3 and SKOV-3 cells were established from ascitic fluid of different ovarian cancer patients, while BG-1 cell line was derived from a poorly differentiated stage III solid primary ovarian tumor. We investigated whether galectin-3 expression of these three commercially available ovarian cell lines (**Figure 1B**) is comparable to the expression of ovarian cell lines established out of primary serous ovarian tumors in our laboratory (KI-OCp1, 11 and 012, stage IIIc) and freshly isolated serous ovarian tumor cells (KI-OCp79, 88 and 91, stage III) (**Figure 1C**). We observed that the different established ovarian tumor cell lines expressed intracellular galectin-3 comparable to freshly isolated ovarian tumor cells.

**Figure 2:**
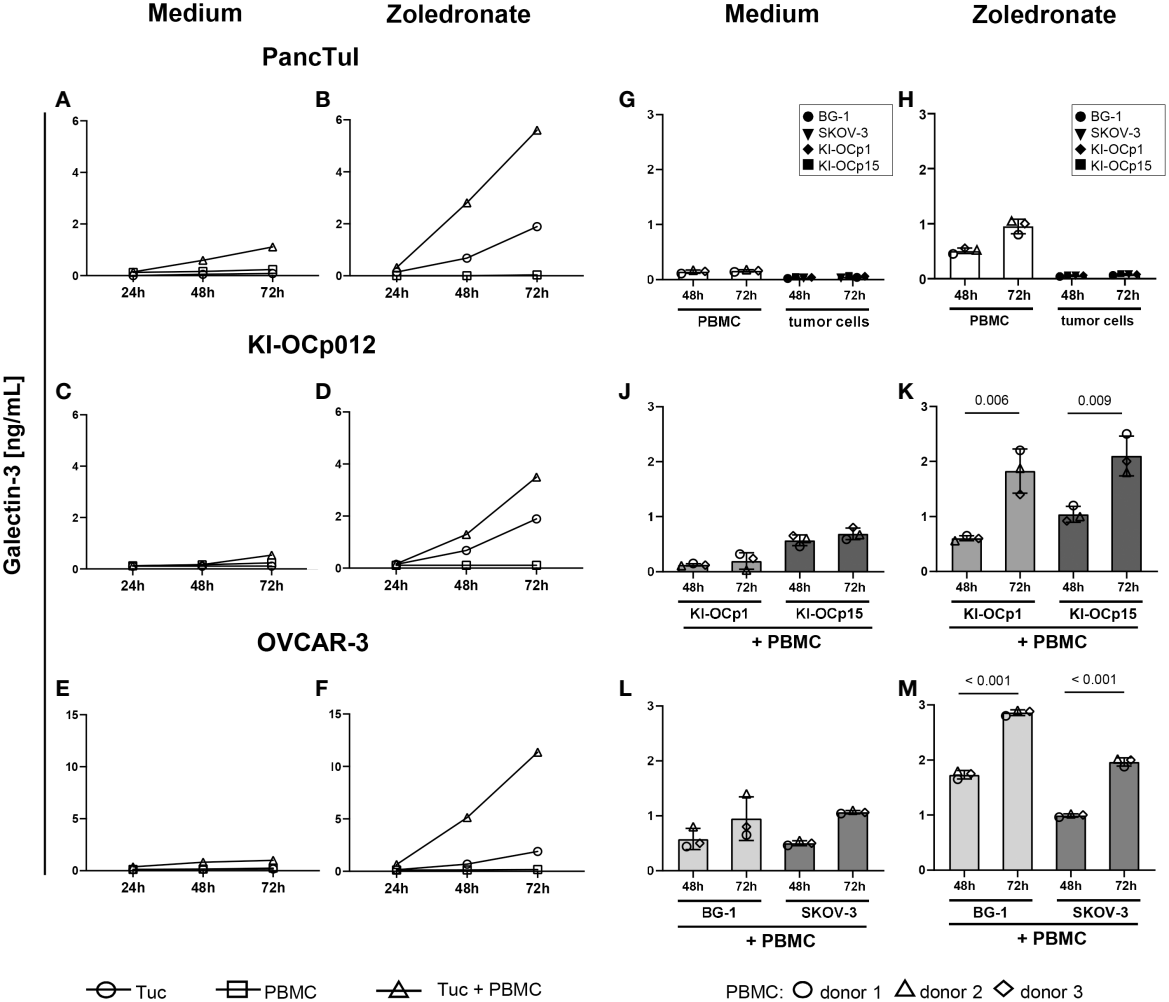
Coculture of PDAC and ovarian cells with zoledronate-activated PBMC induces the highest galectin-3 release. **(A–M)** 3-5x10^5^ freshly isolated PBMC containing 2x10^4^ γδ T cells from four healthy donors were cultured with the indicated tumor cells (Tuc) with a calculated E/T ratio of 1:1 (γδ T cells to tumor cells) or each of them cultured separately. Cells were either cultured in medium **(A, C, E, G, J, L)** or stimulated with 2.5 μM zoledronate **(B, D, F, H, K, M)**. 50 IU/mL rIL-2 was added to each coculture. Cell culture supernatants were collected after 24, 48 and 72 hours and released galectin-3 was determined by ELISA. Statistical comparison was carried out parametrically by using paired, two-tailed *t*-test. Indicated P-values are shown.

As shown by a time kinetic over 72 hours, galectin-3 was released only in small amounts by either pancreatic and ovarian tumor cells (PancTuI, KI-OCp012 or OVCAR-3 cells) or by peripheral blood mononuclear cells (PBMC) (**Figures 2A, C, E**). In contrast, the coculture of the tumor cells combined with the selective activation of Vγ9Vδ2 T cells within the PBMC by zoledronate increased galectin-3 release within 72 hours (**Figures 2B, D, F**). We further compared the commercially available ovarian cell lines BG-1 and SKOV-3 with our established serous ovarian cell line KI-OCp1 and our mucinous ovarian cell line KI-OCp15 by coculturing them with PBMC of three different donors in the absence (**Figures 2G, J, L**) or presence of zoledronate (**Figures 2H, K, M**) for 48 and 72 hours. We confirmed that PBMC and tumor cells released small amounts of galectin-3 (**Figures 2G, H**), which were significantly increased when coculturing PBMC with ovarian cancer cell lines (KI-OCp1 and 15, BG-1 and SKOV-3) in the presence of zoledronate after 72 hours (**Figures 2K, M**). In the absence of zoledronate galectin-3 was slightly increased in the presence of tumor cells after 72 hours compared to 48 hours (**Figures 2J, L**).

The results demonstrated that all analyzed tumor cells express galectin-3 and that the coculture with γδ T cells enhanced the galectin-3 release.

### 3.2 Galectin-3 did not influence the cytotoxic activity of different γδ T-cell subsets

Since an enhanced galectin-3 release is suggested as an intrinsic tumor escape mechanism, we investigated the influence of galectin-3 on the cytotoxicity, proliferation, activation and differentiation of the two major γδ T-cell subsets, Vδ1 and Vδ2 T cells.

**Figure 3:**
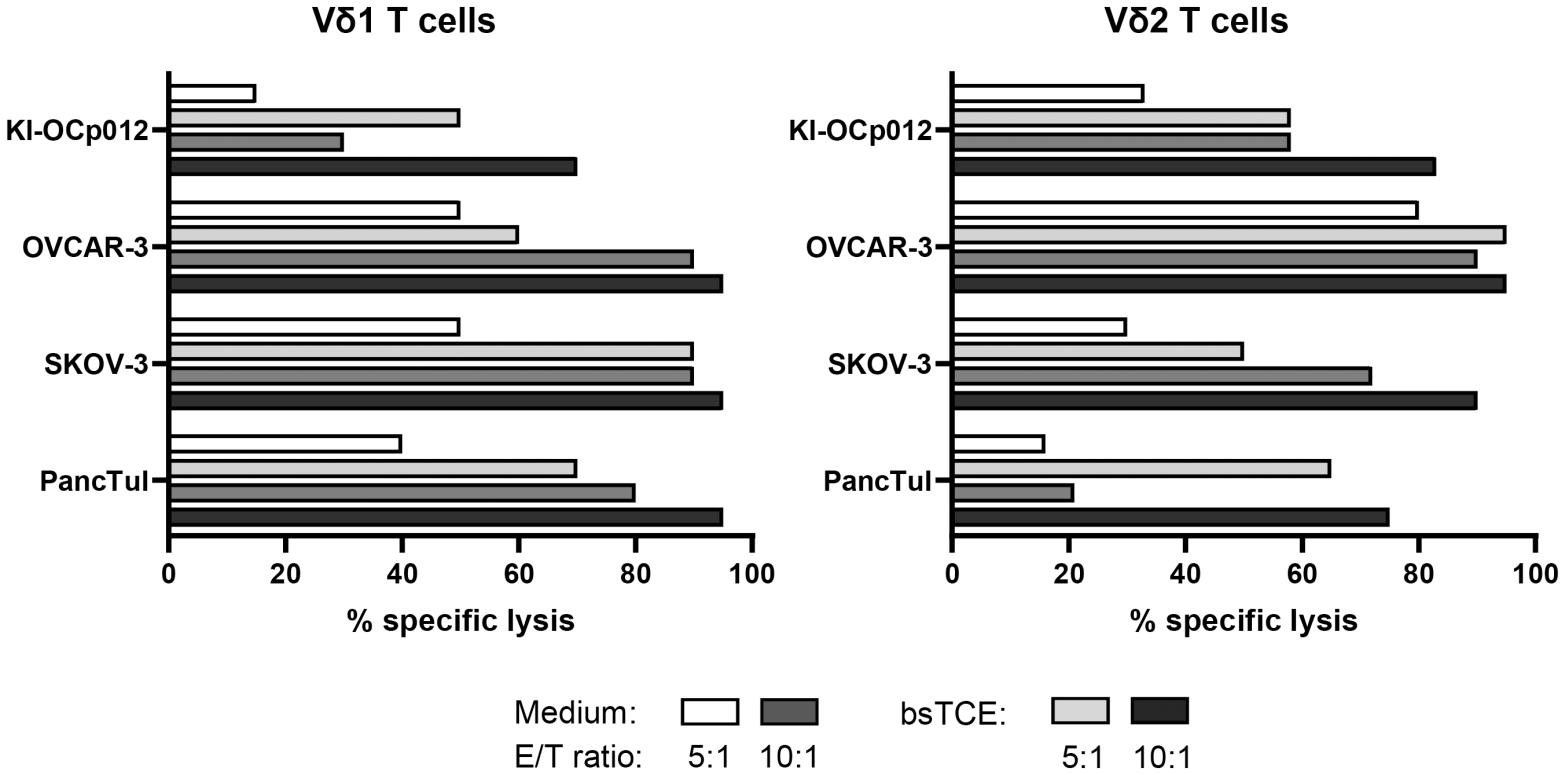
Cytotoxicity of Vδ1 and Vδ2 T cells against tumor cells can be enhanced by bispecific T-Cell Engagers (bsTCE). A total of 10^4^ pancreatic (PancTuI) or ovarian tumor cells (KI-OCp012, OVCAR-3, SKOV-3) per well were cultured in triplicates in complete medium overnight. Impedance of these adherent tumor cells expressed as cell index (CI) was analyzed in 5 minutes steps over ∼24 hours in a RTCA system. After reaching the linear growth phase, tumor cells were cultured with medium alone (spontaneous lysis) or cocultured with Vδ1 and Vδ2 T-cell lines generated out of peripheral blood from one healthy donor. 12.5 IU/mL rIL-2 was added together with Medium (white and middle grey bars) or 1 μg/mL of bsTCE (light and dark grey bars) at an E/T ratio of 5:1 (white and light grey bars) or 10:1 (middle and dark grey bars). The loss of tumor cell impedance and thus a decrease of CI correlated with lysis of tumor cells. Specific lysis of tumor cells was calculated in comparison to spontaneous lysis and maximal lysis (100%) by Triton-X-100 24 hours after adding the γδ T cells.

By firstly focusing on γδ T-cell cytotoxicity, we cocultured a Vδ1 and a Vδ2 T-cell line established from a healthy donor together with ovarian tumor cells (KI-OCp012, OVCAR-3, SKOV-3) and PDAC PancTuI cells in the absence or presence of bispecific T-Cell Engagers (bsTCE) targeting Vδ1 and Vδ2 T cells and HER-2 expressing tumor cells (**Figure 3**). The results revealed that Vδ1 T cells have a superior capacity to lyse SKOV-3 and PancTuI cells, whereas Vδ2 T cells are more effective in killing KI-OCp012 and OVCAR-3. Independently of this observation, the cytotoxic capacity of both γδ T-cell subsets can be increased by an enhanced effector/target ratio (10:1 versus 5:1) and/or the addition of bsTCE (**Figure 3**). Since the lysis of KI-OCp012 and OVCAR-3 differed, we analyzed whether the effector cells or galectin-3 release is crucial for the difference in lysis.

**Figure 4:**
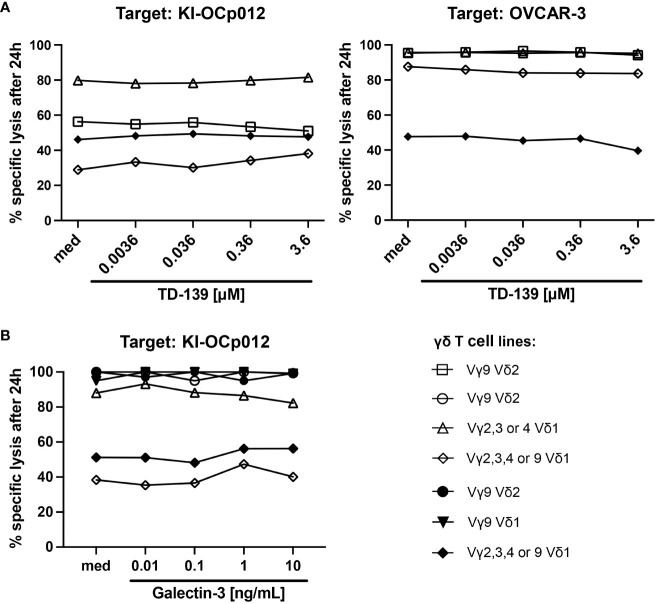
Galectin-3 does not influence γδ T-cell cytotoxicity against ovarian tumor cells. **(A, B)** A total of 10^4^ indicated ovarian tumor cells per well were cultured in triplicates in complete medium overnight. Impedance of these adherent tumor cells expressed as cell index (CI) was analyzed in 5 minutes steps over ∼24 hours in a RTCA system. After reaching the linear growth phase, tumor cells were cultured with medium (spontaneous lysis) or cocultured with different γδ T-cell subset lines isolated out of peripheral blood from healthy donors (open symbols, n = 4) or ovarian cancer patients (closed symbols, n=3) at an E/T ratio of 5:1. The Vδ1 T-cell line marked with a closed rhombus is autologous to KI-OCp012 tumor cells. 12.5 IU/mL rIL-2 was added to the cultures and cells were stimulated with 1 μg/mL of bsTCE in the absence (med) or presence of the indicated concentrations of galectin-3 inhibitor TD-139 **(A)** or galectin-3 in distinct concentrations (0.01, 0.1, 1, 10 ng/mL) **(B)**. The loss of tumor cell impedance and thus a decrease of CI correlated with lysis of tumor cells. Lysis of tumor cells was measured after normalization to 1 in 3 minutes steps for additional 24 hours and compared to maximal lysis (100%) by Triton-X-100. Specific lysis of tumor cells was calculated in comparison to spontaneous lysis and maximal lysis (100%) by Triton-X-100 24 hours after adding the γδ T cells.

Therefore, we generated diverse γδ T-cell lines from PBMC of healthy donors and ovarian cancer patients as well as an autologous one and investigated their efficacy to lyse these both ovarian tumor cells (**Figure 4**). The γδ T-cell cytotoxicity of Vδ1 as well as of Vδ2 T cells was impaired against KI-OCp012 cells compared to OVCAR-3 cells (**Figure 4A**, med). Obviously, the Vδ1 T-cell line generated out of patient OCp012 has a low cytotoxicity against the autologous KI-OCp012 cells and the allogeneic OVCAR-3 cells. Besides, the addition of different concentrations of TD-139, a potent small-molecule inhibitor of galectin-3, did not improve γδ T-cell cytotoxicity against ovarian cancer cells after 24 hours (**Figure 4A**) or at earlier time points (**Supplementary Figure 2**).

The results were substantiated by experiments adding different galectin-3 concentrations (ranging from 0.01 to 10 ng/mL) to the cocultures (**Figure 4B**). Our results revealed that the cytotoxic capacity of the γδ T cell lines generated out of PBL or TIL is very similar in the absence of an immunosuppressive tumor microenvironment. Further, the different galectin-3 concentrations did not influence the γδ T-cell-mediated cytotoxicity towards the ovarian cancer cells after 24 hours (**Figure 4B**) or after earlier time points (**Supplementary Figure 3**).

In sum, comparable to Vδ2 T cells, cytotoxicity of Vδ1 T cells against tumor cells is not influenced by galectin-3.

### 3.3 Proliferation of Vδ2 T cells but not of Vδ1 T cells was inhibited by galectin-3 producing ovarian tumor cells

Previously, we found that galectin-3 released from PDAC cells inhibits Vδ2 T-cell proliferation (18). Therefore, we asked whether other tumor entities such as ovarian cancer cells have the same capacity, and whether Vδ1 T-cell proliferation is influenced by galectin-3.

**Figure 5:**
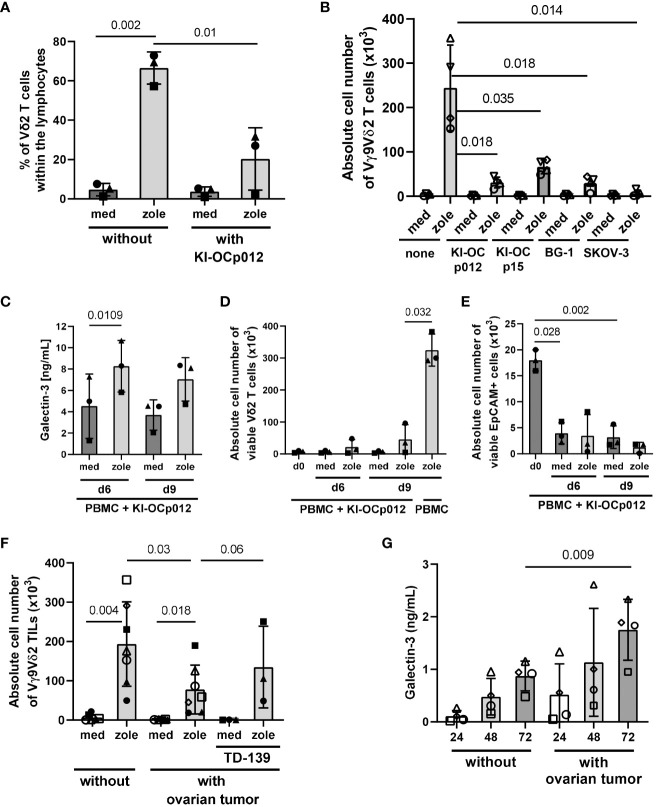
Coculturing ovarian cancer cells with PBMC or TIL leads to a decreased Vδ2 T-cell proliferation and increased amounts of released galectin-3. A total of 3-7x10^5^ freshly isolated PBMC from healthy donors (each n = 3 in **(A)** and **(C–E)**), (n = 4 in **(B)**) and 1.5-7x10^5^ freshly isolated TIL from an ovarian cancer patients (n = 7 in **(F, G)**) were cultured for 6 **(C–E)**; 7 **(F, G)** or 9 days **(A–E)** with or without KI-OCp012 **(A–E)**, the indicated tumor cells **(B)** or autologous tumor cells **(F, G)**. The E/T ratio was 1:1 calculated with 2×10^4^ γδ T cells within PBMC or TIL and the same amount of tumor cells. **(A–G)** Cells were either cultured in medium or stimulated with 2.5 μM zoledronate. 50 IU/mL rIL-2 was added to the PBMC or TIL. **(F)** 100 nM of the galectin-3 inhibitor TD-139 was added daily as indicated. Proliferation of Vδ2 T cells was measured and expressed **(A)** in percentage after 9 days or **(B, D, F)** the absolute cell number ± SD was determined after the 9 days **(B)**, the indicated time points **(D)** or after 7 days **(F)**. **(E)** After 6 and 9 days, absolute cell number ± SD of EpCAM-expressing ovarian tumor cells was measured by LSR-Fortessa. **(C, G)** Cell culture supernatants were collected from coculturing **(C)** PBMC or **(G)** TIL with allogeneic or autologous tumor cells, respectively, after 24, 48 and 72 hours and released galectin-3 ± SD was determined by ELISA. Statistical comparison was carried out parametrically by using paired, two-tailed *t*-test. Indicated P-values are shown.

We determined the percentage (**Figure 5A**) or the absolute cell number of Vδ2 T cells (**Figure 5B**) within PBMC and added a specific amount of PBMC to the culture to provide an E/T ratio of 1:1 of Vδ2 T cells and tumor cells. After 9 days of coculture, we analyzed the percentage (**Figure 5A**) or absolute cell number of viable proliferating Vδ2 T cells (**Figure 5B**) and tumor cells again. As a control PBMC were cocultured without tumor cells (**Figures 5A, B**; without KI-OCp012 or none). A vigorous selective Vδ2 T-cell growth after stimulation with zoledronate in the absence of the indicated ovarian tumor cells compared to the control was observed after 9 days (**Figures 5A, B**). In the presence of ovarian tumor cells (KI-OCp012, KI-OCp15, BG-1, SKOV-3), the proliferation of Vδ2 T cells was significantly inhibited after stimulation with zoledronate in comparison to cultures without tumor cells (**Figures 5A, B**).

An increased release of galectin-3 was observed when coculturing the PBMC with KI-OCp012 cells after stimulation with zoledronate for 6 to 9 days (**Figure 5C**). A decrease of the absolute cell number of viable Vδ2 T cells in the presence of ovarian tumor cells together with zoledronate is shown for these two time points (**Figure 5D**). In contrast, a 46-fold increase of Vδ2 T cells within the PBMC in the absence of ovarian tumor cells is demonstrated in the same figure. In parallel, the absolute cell number of viable EpCAM (CD326)-positive ovarian tumor cells is reduced compared to day 0 (**Figure 5E**), which underline the observation that Vδ2 T-cell cytotoxicity is not influenced by galectin-3 (**Figure 4**).

Following the assumption that Vδ2 TIL are in a pre-activated stage, we analyzed the proliferative capacity of freshly isolated Vδ2 TIL cocultured in medium without or with freshly isolated autologous ovarian tumor cells (E/T ratio 1:1) in further experiments. Comparable to PBMC, an inhibition of the Vδ2 T-cell outgrowth was observed after stimulation with zoledronate in the presence of autologous ovarian tumor cells in comparison to the absence of tumors. This is shown for the absolute cell number of viable Vδ2 TIL of seven different donors (**Figure 5F**). The daily supplementation of galectin-3 inhibitor TD-139 to three different patients (closed symbols) restored the tumor cell mediated inhibition of Vδ2 T-cell proliferation (**Figure 5F**). An increase of galectin-3 was measured when Vδ2 TIL were cocultured with freshly isolated autologous ovarian tumor cells compared to the culture without tumor cells (**Figure 5G**).

**Figure 6:**
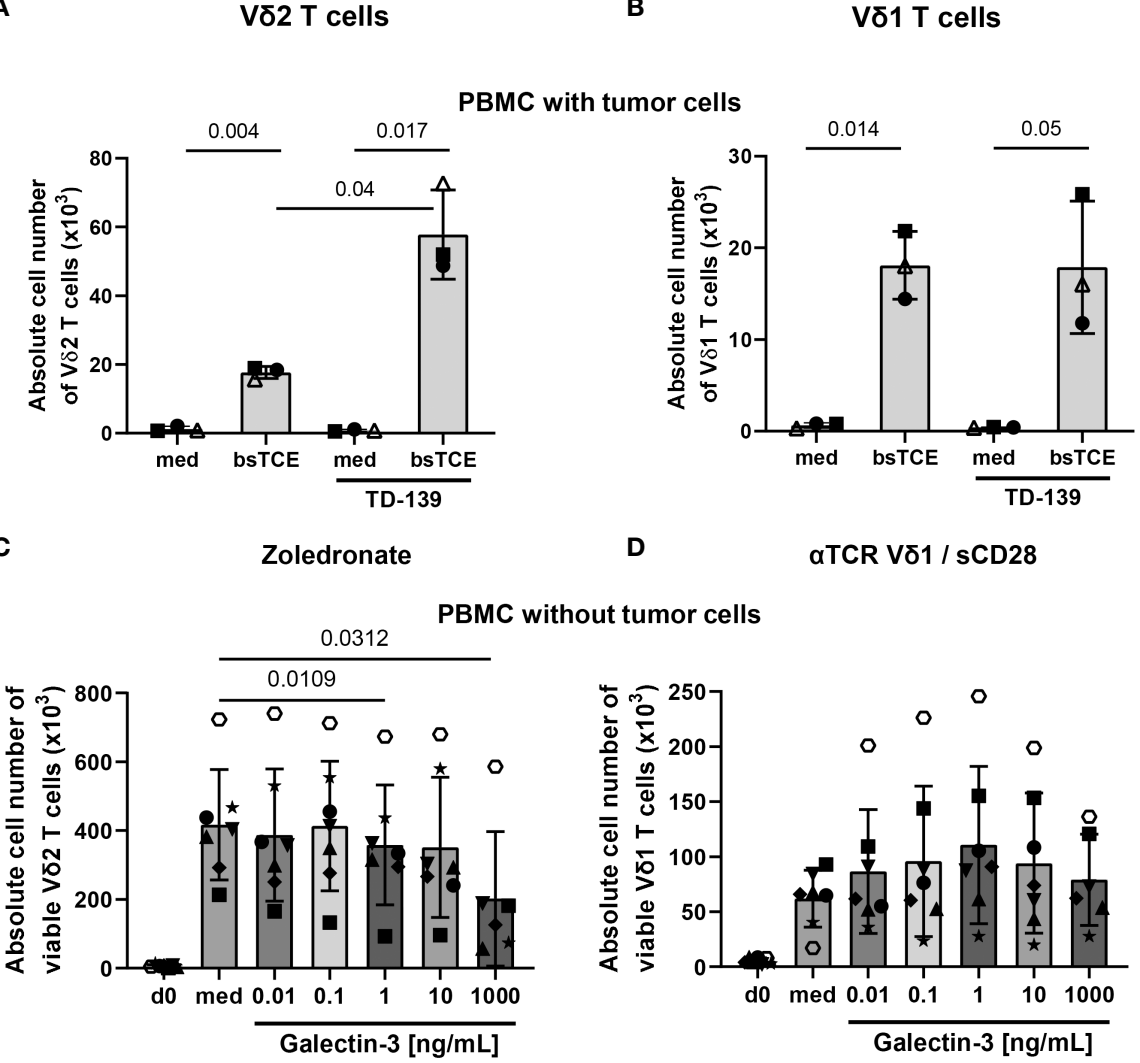
Different effects of galectin-3 on Vδ1 and Vδ2 T cells. **(A-D)** A total of 3-7x10^5^ freshly isolated PBMC from healthy donors (closed symbols, n = 2-6) or from ovarian cancer patients (open symbols, n =1) were either stimulated **(A)** with 1 µg/mL bsTCE targeting Vδ2 T cells, **(B)** with 1 µg/mL bsTCE targeting Vδ1 T-cells, **(C)** with 2.5 μM zoledronate for Vδ2 T-cell proliferation or **(D)** plate-coated anti-TCR Vδ1 and soluble CD28 mAb for Vδ1 T-cell proliferation (each 2x10^4^ γδ T cells per well). 50 IU/mL rIL-2 was added to the PBMC and either complete medium (med) or **(A, B)** supplemented daily with 100 nM galectin-3 inhibitor TD-139 or **(C, D)** distinct concentrations of galectin-3 (0.01, 0.1, 1, 10 and 1000 ng/mL). After 9 days absolute cell number ± SD of viable Vδ2 and Vδ1 T cells was measured by LSR-Fortessa. Statistical comparison was carried out parametrically by using paired, two-tailed *t*-test or non-parametrically by using a Wilcoxon matched-pairs signed rank test. Indicated P-values are shown.

Since the antigens for other γδ T-cell subsets than Vδ2 T cells are not well defined, we used plate-coated anti-TCR Vδ1 mAb together with soluble anti-CD28 mAb to stimulate Vδ1 T cells within PBMC and TIL in several of the experiments. However, the coating of plates with anti-TCR Vδ1 mAb was not possible when coculturing PBMC or TIL with adherent tumor cells. Therefore, we stimulated Vδ1 and Vδ2 T cells with our bsTCE. These both bsTCE selectively stimulated the different γδ T-cell subsets within PBMC or TIL and significantly enhanced the γδ T-cell mediated lysis against tumor cells (3) (manuscript in preparation). Since these bsTCE are not developed to induce γδ T-cell proliferation, our results with bsTCE stimulation revealed only a slight proliferation of Vδ1 or Vδ2 T cells within PBMC (closed symbols) or TIL (open symbol, autologous situation) cocultured with freshly isolated ovarian tumor cells (KI-OCp79, 88 and 91). Nevertheless, the proliferation was enough to determine a different effect of the galectin-3 inhibitor TD-139 on Vδ1 versus Vδ2 T cells. While the daily supplementation of TD-139 over 9 days of culturing restored the tumor cell mediated inhibition of Vδ2 T-cell proliferation, Vδ1 T cells are not influenced by TD-139 (**Figures 6A, B**).

To test whether the concentration of galectin-3 released by the freshly isolated tumor cells was not sufficient to inhibit Vδ1 T-cell proliferation, we added different concentrations of galectin-3 to PBMC either stimulated with zoledronate or anti-TCR Vδ1/anti-CD28 mAbs as illustrated in **Figures 6C, D**. Vδ2 and Vδ1 T cells expanded 9 days after their selective activation compared to day 0. After stimulation, Vδ2 T cells expanded by 45-fold and Vδ1 T cells by 11-fold increase (**Figures 6C, D**). While the addition of increasing concentrations of galectin-3 inhibited Vδ2 T-cell proliferation, the Vδ1 T-cell proliferation was not impaired and slightly enhanced in the presence of 1-10 ng/mL recombinant galectin-3 (**Figures 6C, D**).

Taken together, galectin-3 inhibits the Vδ2 T-cell proliferation but not the Vδ1 T-cell proliferation.

### 3.4 Different effects by galectin-3 on the differentiation and activation of Vδ1 versus Vδ2 T cells

Since we observed different effects of galectin-3 on the proliferation of Vδ1 and Vδ2 T cells, we asked whether other features such as differentiation, activation and expression of immune check point markers differ between Vδ1 and Vδ2 T cells after their exposure to galectin-3.

**Figure 7:**
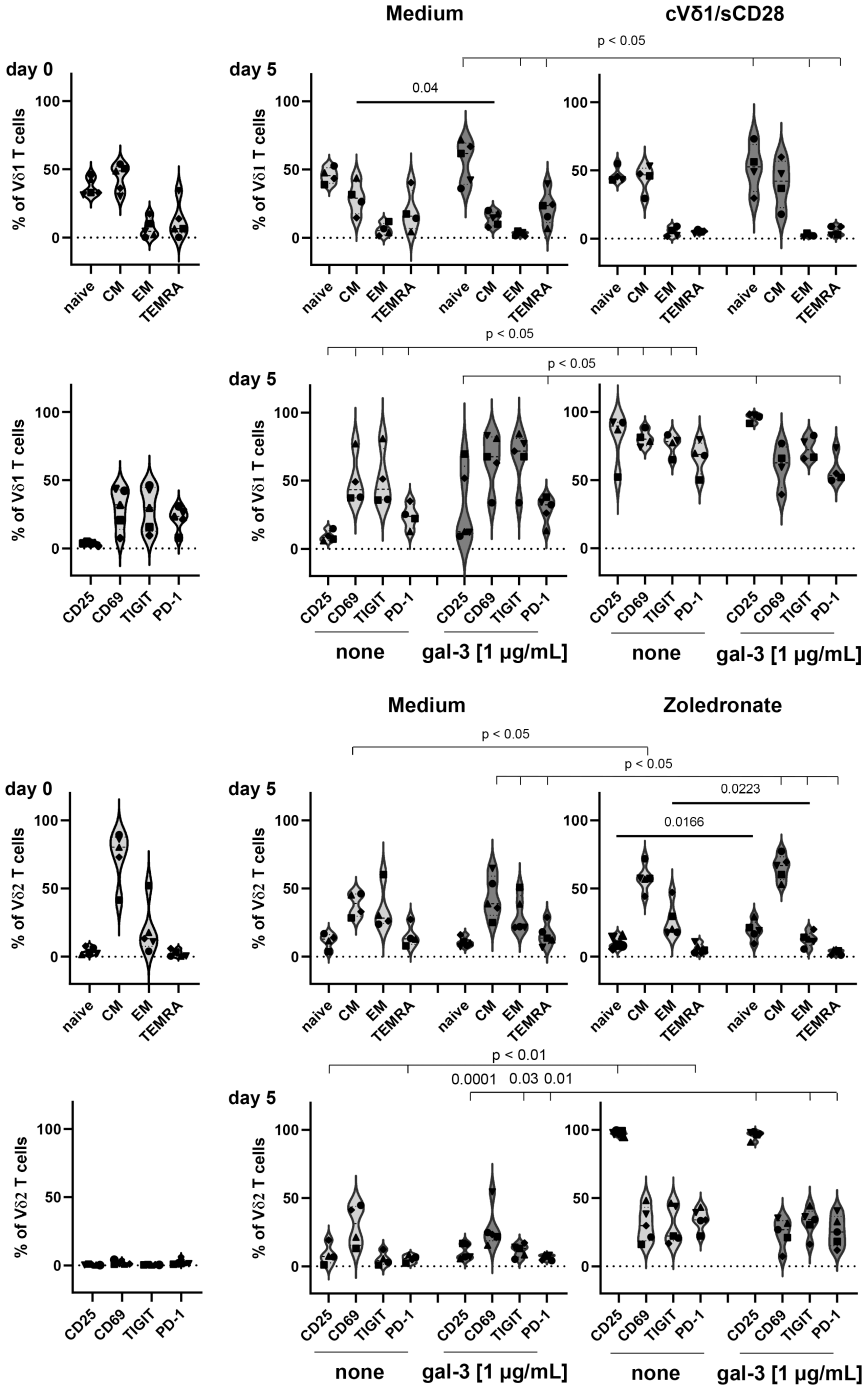
Expression of differentiation, activation and immune check point markers on Vδ1 and Vδ2 T cells. 5x10^5^ PBMC (n =5) were stained with anti-CD45RA and anti-CD27 mAbs to determine naïve, central and effector memory (CM and EM) or TEMRA cells of Vδ1 and Vδ2 T cells at day 0. Activation (CD69 and CD25) and immune check point (TIGIT and PD-1) markers were analyzed at day 0. Residual cells (5x10^5^ cells/well) were cultured in complete medium, stimulated with 2.5 μM zoledronate or with 10 μg/mL coated anti-Vδ1 and 1 μg/mL soluble anti-CD28 mAbs. Medium or 1 µg/mL galectin-3 (gal-3) was added as indicated. After 5 days, cells were stained with the same mAbs as on day 0 and measured by LSR-Fortessa. A gate was set on CD45, CD3, TCRγδ and Vδ1 or Vδ2 T cells to determine naïve, CM, EM T cells and TEMRA cells and the activation and immune check point markers on both γδ T-cell subsets. Statistical comparison was carried out parametrically by using paired, two-tailed *t*-test or non-parametrically by using a Wilcoxon matched-pairs signed rank test. Indicated P-values are shown.

Vδ1 T cells initially (d0) comprised less central memory (CM) T cells and more T effector memory cells re-expressing CD45RA (TEMRA cells) than Vδ2 T cells from the same donors (**Figure 7**, d0). The expression of the activation marker CD69 and of immune check point T cell immunoreceptor with Ig and ITIM domains (TIGIT) and programmed cell death protein (PD)-1 was initially enhanced on Vδ1 T cells in comparison to Vδ2 T cells (**Figure 7**, d0). After culturing the Vδ1 T cells in 1 µg/mL galectin-3 for 5 days, the percentage of Vδ1 CM T cells was significantly diminished and the expression of CD25, CD69, TIGIT and PD-1 was increased compared to the culture without galectin-3. This was concentration dependent, since Vδ1 T cells were activated with high galectin-3 concentrations (**Figure 7**, Medium) but not with low galectin-3 concentrations (**Supplementary Figure 4**, Medium). In contrast, Vδ2 T cells were not affected by any galectin-3 concentration (**Figure 7**; **Supplementary Figure 4**, Medium).

The stimulation of Vδ1 T cells with coated anti-Vδ1 and soluble anti-CD28 mAb and of Vδ2 T cells with zoledronate, induced significant alterations in the differentiation status and an enhanced expression of activation and immune check point markers. More importantly, only the combination of zoledronate and 1 µg/mL galectin-3 stimulation, significantly enhanced the percentage of naïve Vδ2 T cells and significantly reduced the percentage Vδ2-expressing effector memory (EM) T cells (**Figure 7**; **Supplementary Figure 4**, right panel).

A reduction of Vδ2-expressing effector memory cells after zoledronate stimulation together with 1 µg/mL galectin-3 could explain the significant galectin-3 mediated reduction of Vδ2 T-cell proliferation.

**Figure 8:**
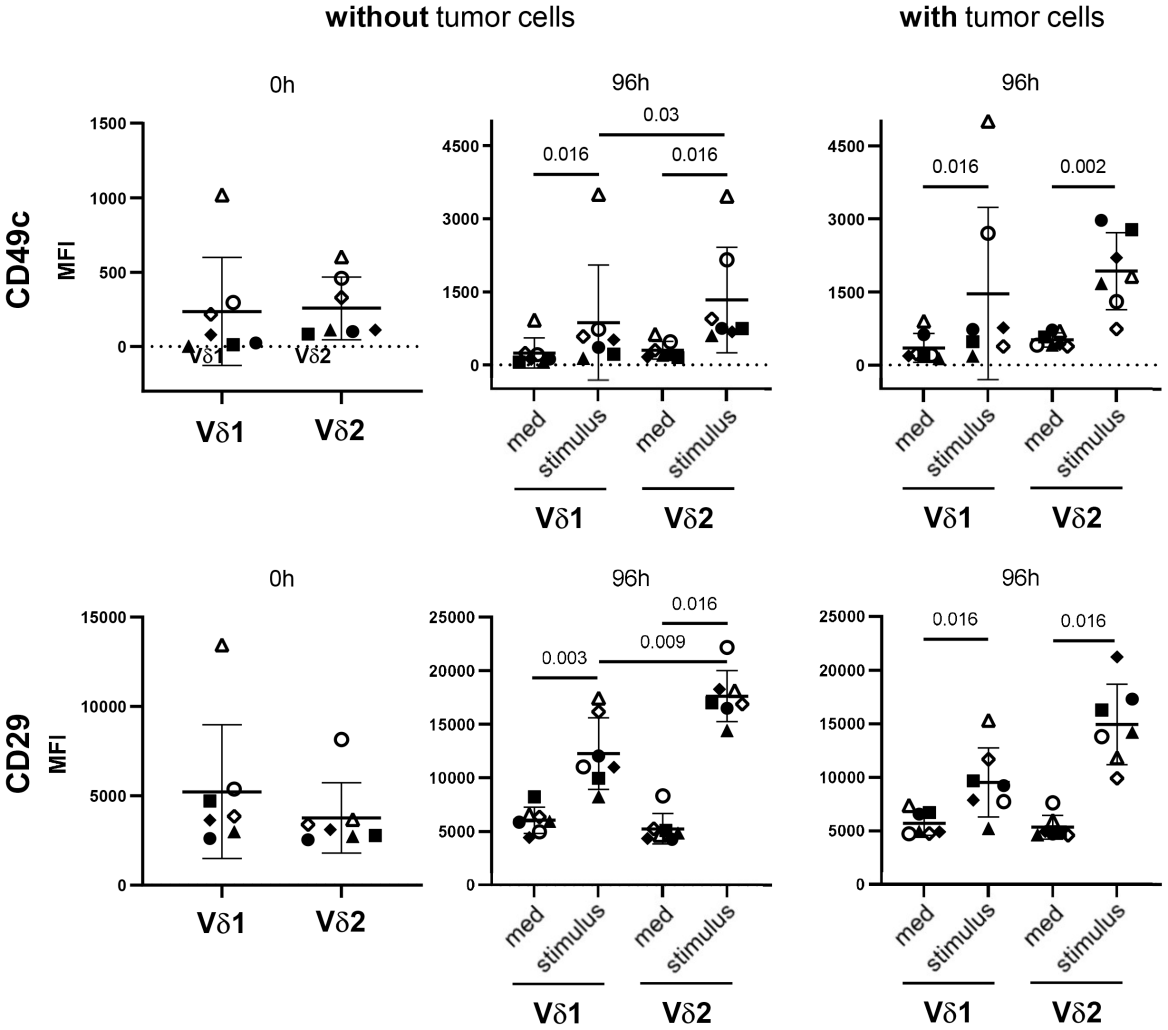
Differential expression of CD49c/CD29 on Vδ1 and Vδ2 T cells. 5x10^5^ PBMC (closed symbols, n =4) and TIL (open symbols, n = 3) were stained after isolation (0 h) with anti-CD49c and anti-CD29 mAb. Residual cells (5x10^5^ cells/well) (without tumor cells) were cultured in complete medium, stimulated with 2.5 μM zoledronate or with coated anti-Vδ1 mAb (10 μg/mL) together with soluble anti-CD28 mAb (1 μg/mL) (stimulus). In parallel, 5x10^5^ PBMC (closed symbols) or TIL (open symbols) were co-cultured with 5x10^4^ OVCAR-3 cells (with tumor cells) in the presence of bispecific T-Cell Engagers (stimulus) selectively targeting HER-2 expressing ovarian tumor cells to Vγ9Vδ2 or Vδ1 T cells. After 96 hours, cells were stained and measured by LSR-Fortessa. A gate was set on CD45, CD3, TCRγδ and Vδ1 or Vδ2 T cells to determine the CD49c and CD29 expression on both γδ T-cell subsets after 0 and 96 hours (h). Statistical comparison was carried out parametrically by using paired, two-tailed *t*-test or non-parametrically by using a Wilcoxon matched-pairs signed rank test. Indicated P-values are shown.

A further explanation for the differential sensitivity of the γδ T-cell subsets towards galectin-3 can be found in the expression of the galectin-3 binding partner. Our previous results suggest that the binding of galectin-3 to α3β1 integrin prevents the proliferation-promoting effect of CD49c/CD29 on Vδ2 T cells (18). Before stimulation, CD49c and not CD29 is nearly similar expressed on Vδ2 T cells compared to Vδ1 T cells determined within PBMC (closed symbols) and TILs (open symbols) (**Figure 8**, 0 h). After stimulation, CD49c and CD29 are significantly upregulated in Vδ1 T cells and in Vδ2 T cells. However, an up-regulation of both integrins was more pronounced in Vδ2 T cells than in Vδ1 T cells, and significant in the absence of tumor cells. The superior expression of CD49c and CD29 on Vδ2 T cells compared to Vδ1 T cells is shown already after 20 hours of stimulation (**Supplementary Figure 5**) and is further increased 96 hours after stimulation (**Figure 8**).

The enhanced CD49c and CD29 expression on Vδ2 T cells after activation explain the different susceptibility of Vδ2 T cells to galectin-3.

## 4 Discussion

This study demonstrated that the coculture of stimulated γδ T cells with different tumor cells significantly enhanced the galectin-3 release, which did not influence γδ T-cell cytotoxicity against tumor cells. More importantly, the Vδ2 T-cell proliferation was inhibited in the presence of galectin-3, whereas the Vδ1 T-cell proliferation was slightly increased. The data are of great interest for an *in vivo* application of Vδ2 T-cell stimulating antigens such as zoledronate, which induces a selective Vδ2 T-cell outgrowth. A main problem of the repetitive *in vivo* application of zoledronate together with rIL-2 is the exhaustion of the Vδ2 T cells (31–33). Our data suggests that Vδ2 T cells infiltrating in tumors are inhibited in their proliferation if galectin-3 concentrations are increased since activation of Vδ2 TIL cocultured with tumor cells inhibited Vδ2 T-cell expansion and reduced effector memory activation. Moreover, Vδ2 T cells within PBMC, which grow out selectively after stimulation with zoledronate and can migrate to the tumor site, can also be inhibited in their proliferation after cross talk with tumor cells. Beside tumor cells, other cells in the immunosuppressive tumor microenvironment (TME) produce galectin-3. For instance, in a lung adenocarcinoma tumor sphere-model, which mimic an immunosuppressive TME, galectin-3 is released in the TME and modulated the tumor infiltrating immune cells such as regulatory T cells (Treg) (34). These authors demonstrated that the patients with high soluble galectin-3 levels had more Treg cells, which can inhibit T cells (34). Treg are also suggested to inhibit γδ T-cell proliferation (35). In addition, galectin-3 is described to advance macrophage infiltration, M2-polarization and immunosuppressive effects of myeloid derived suppressor cells and Treg on cytotoxic CD8 T cells (36).

Our previous results demonstrated that the galectin-3 binding to glycosylated α3β1 integrin (CD49c/CD29) prevents the Vδ2 T-cell proliferation-promoting effect of CD49c/CD29. Since Vδ1 T cells are enriched at the tumor site of pancreatic and ovarian tumor cells (3, 18), the different impact of galectin-3 on Vδ1 T cells is of high interest and makes them attractive for γδ T-cell-based immunotherapy. One explanation for the different susceptibility to galectin-3 treatment of Vδ1 and Vδ2 T cells is due to the lower expression of CD49c/CD29 on Vδ1 T cells compared to Vδ2 T cells after their activation. CD49c/CD29 expressed on endothelial cells is described to bind galectin-3 producing metastatic cells thereby stabilizing tumor/endothelial cell adhesion (37). Chen and colleagues reported that type I collagen (Col1) homotrimer is produced by pancreatic cancer cells and binds to α3β1 integrin thereby promoting oncogenic signaling and cancer cell proliferation. The deletion of Col1 homotrimers increases T-cell infiltration and improved anti-PD-1 immunotherapy (38). Tribulatti and colleagues demonstrated that galectin-3 impaired antigen-specific T-cell responses in murine CD4 T cells (39). Others demonstrated that galectin-3 is an inhibitory regulator also of human conventional T-cell activation and promotes TCR down-regulation, failure of TCR and CD8 colocalization and T-cell anergy (19, 40, 41). While an exact role of CD49c/CD29 interaction with galectin-3 is not described in human CD4 and CD8 αβ T cells, these interaction partners are responsible for the failure of Vδ2 T-cell proliferation in the presence of galectin-3 producing cells such as tumor cells. CD49c/CD29 is already described to be expressed on γδ T cells (42). Additionally, our results revealed an obvious and significant difference between Vδ2 and Vδ1 T cells after their activation which explains the different susceptibility of galectin-3 on the proliferation of these γδ T-cell subsets.

An increased Vδ1 T-cell infiltration in tumor tissue compared to the blood in ovarian cancer patients is described by us (3) and other groups (43–46). Intra-tumoral CD73-expressing Vδ1 TIL in breast cancer patients are suggested to have immunoregulatory properties which often suppress anti-tumor response (47). As shown in **Figure 7**, Vδ1 T cells isolated of PBMC from healthy donors or ovarian cancer patients are mainly naïve, CM, TEMRA T cells, which highly expressed immune check point inhibitors and exhaustion markers such as TIGIT and PD-1. Upon activation of the Vδ1 T cells, the CM T-cell population increased after 5 days (**Figure 7**) and EM population after 14 days (data not shown). This is in line with our own unpublished data demonstrating that the percentage of CM and EM Vδ1 TIL is increased. The expression of PD-1 and TIGIT on Vδ1 TIL is drastically enhanced compared to Vδ2 T cells generated out of blood or tumor tissue (**Figure 7** and unpublished data). However, PD-1 and TIGIT are transiently increased in zoledronate or bsTCE activated Vδ2 T cells after 5 days (**Figure 7**) and decreased after 14 days (data not shown). Weimer and colleagues demonstrated an exhausted phenotype of Vδ1 TIL in ovarian cancer patients (45). On Vδ1 TIL, PD-1 was increased on CM, while ectonucleoside triphosphate diphosphohydrolase-1 (CD39) was enhanced on EM. Interestingly, ecto-5´-nucleotidase (CD73) was not expressed on ovarian γδ TIL (45). CD39 and CD73 are enzymes which mediate a gradual hydrolysis of danger signals of ATP and ADP to anti-inflammatory adenosine, which induce exhaustion of cells (48–50).

Although Vδ1 TIL seem to be in an exhausted stage in several advanced ovarian cancer patients, we were able to stimulate and expand Vδ1 TIL and PBMC in the absence of autologous tumor cells. After expansion, Vδ1 T cells cocultured with ovarian cancer cells exert a high cytotoxicity which was not influenced by galectin-3 release of tumor cells. In addition, Vδ1 T-cell proliferation was not influenced by galectin-3 which is probably an advantage for Vδ1 T-cell based immunotherapy. Since Vδ2 T cells are the predominant γδ T-cell subset in the blood of Caucasian population, in contrast to Asian and African population, almost all human γδ T-cell research is focused on Vδ2 T cells. However, Fisher and colleagues demonstrated that Vδ1 T cells and Vδ1/Vδ2-negative T cells within PBMC possess many characteristics, which recommend them for T-cell based immunotherapy instead of Vδ2 T cells. These characteristics include an enhanced cytotoxic activity of Vδ1 T cells per se, a reduced differentiation to a CD27, CD45RA and CD62L pattern, a long persistence in patients and a decreased PD-1 expression after their activation (51). Here, we described a resistance of Vδ1 T cells against galectin-3 mediated inhibition of proliferation, which is regarded as an additional advantage for Vδ1 T-cell-based immunotherapy. In addition, we observed an enhanced percentage of ovarian Vδ1 TIL coexpressing Vγ9 and expressing PD-1 (unpublished observation). These cells are EM Vδ1 TIL with a high cytotoxic activity towards different ovarian cancer cells (**Figure 7**). The enhanced cytotoxic activity was supported by the slight expression of PD-L1 on ovarian cancer cells (3) suggesting that a certain Vδ1 T cell-subset could be suitable for a Vδ1 T-cell based immunotherapy.

## Data availability statement

The original contributions presented in the study are included in the article/**Supplementary Material**. Further inquiries can be directed to the corresponding author.

## Ethics statement

The studies involving humans were approved by Ethic Committee of the Medical Faculty of the CAU Kiel, code number: D 445/18. The studies were conducted in accordance with the local legislation and institutional requirements. The participants provided their written informed consent to participate in this study.

## Author contributions

JS: Data curation, Investigation, Methodology, Visualization, Writing – original draft, Writing – review & editing. H-HO: Conceptualization, Investigation, Methodology, Supervision, Visualization, Writing – review & editing. MP: Resources, Writing – review & editing. NH: Methodology, Resources, Writing – review & editing. WS: Funding acquisition, Resources, Writing – review & editing. DB: Resources, Supervision, Writing – review & editing. DW: Conceptualization, Funding acquisition, Investigation, Methodology, Project administration, Supervision, Writing – original draft, Writing – review & editing.
