# Supplementary material for: Vdelta1 T cells are more resistant than Vdelta2 T cells to the immunosuppressive properties of galectin-3
Source: Front Immunol. 2024 Jan 8;14:1286097. doi: 10.3389/fimmu.2023.1286097 (PMC10800970; doi:10.3389/fimmu.2023.1286097)
Supplement: Supplementary file 1 [file DataSheet_1.pdf]

Supplemental Fig. 1

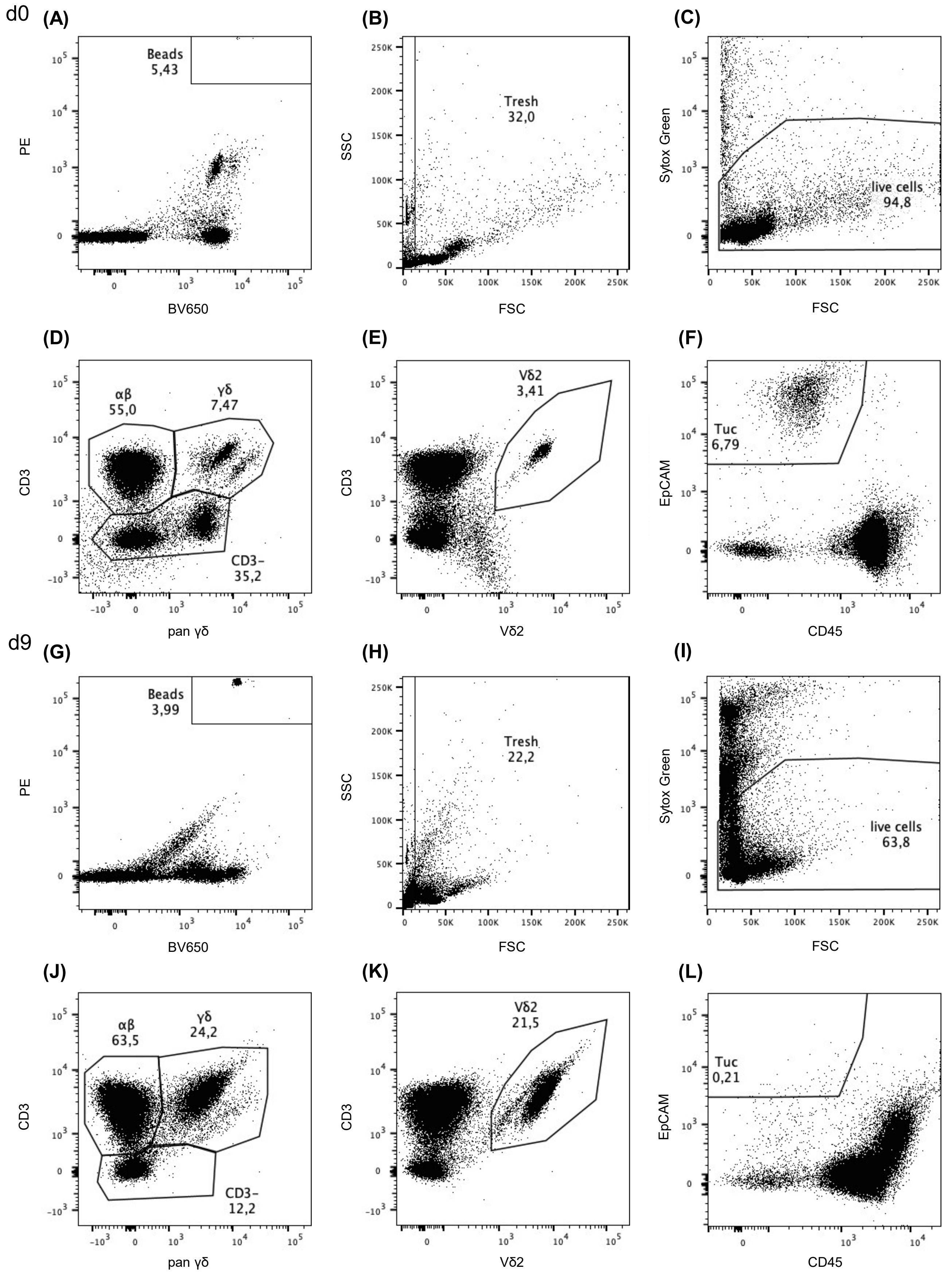

Suppl. Fig. 1 Gating strategy for absolute cell number analysis. **(A, G)** For absolute cell number calculations, a defined number of beads was measured with PE against BV650. **(B, H)** The threshold was set using forward scatter (FSC) to eliminate any kind of disturbing signals. **(C, I)** Within the threshold gating, Sytox Green against FSC was used to get rid of dead cells and include only viable cells in the analysis. Afterwards, the different cell populations were distinguished within the live cells: **(D, J)** CD3 against TCR pan γδ for different lymphocyte populations, **(E, K)** CD3 against Vδ2 for the Vδ2 T-cell subset and **(F, L)** EpCAM against CD45 for the tumor cells.

Supplemental Fig. 2

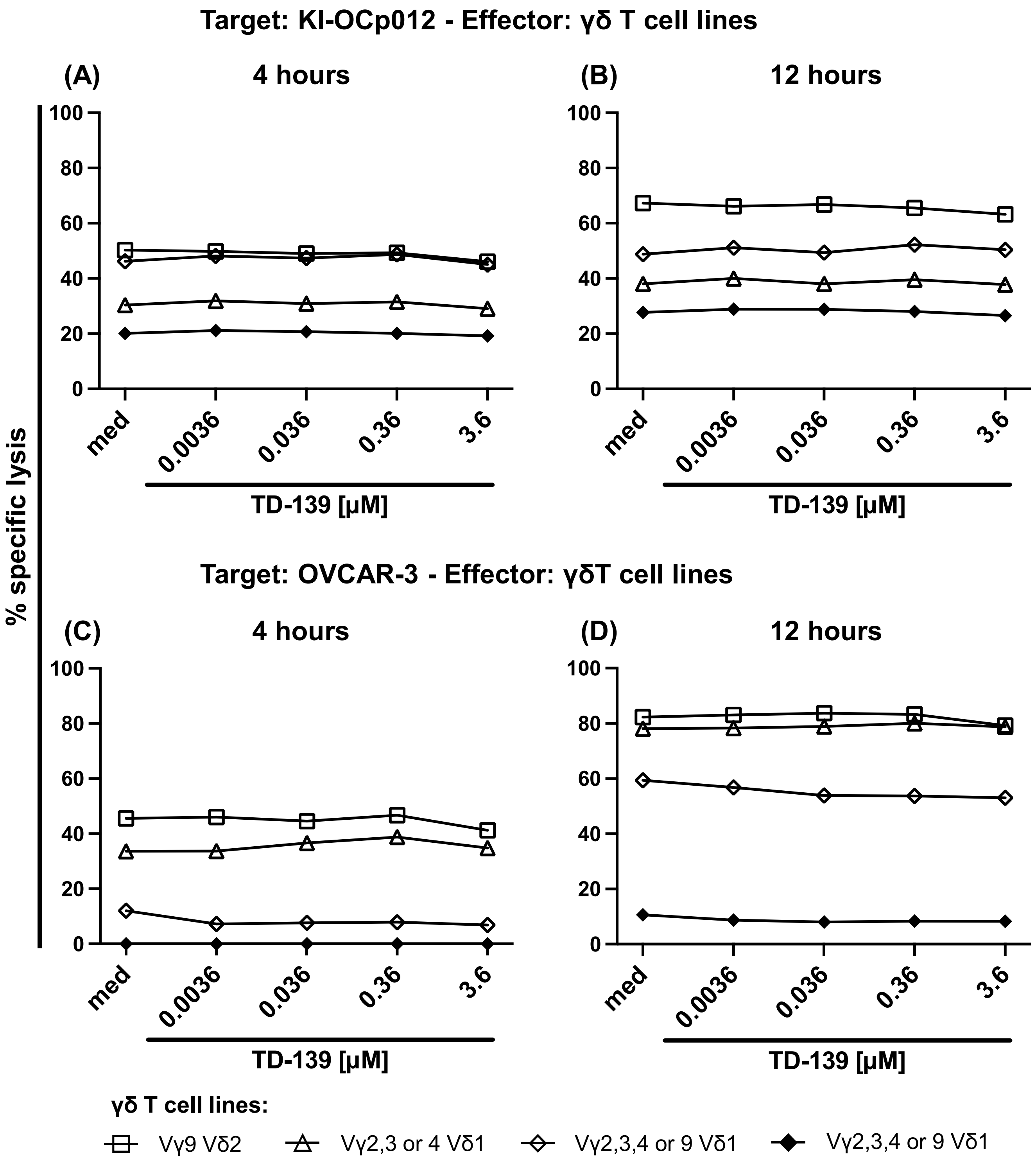

Suppl. Fig. 2 Blocking galectin-3 does not influence γδ T-cell cytotoxicity against ovarian cancer cells. (**A-D**) A total of 10^4 indicated ovarian tumor cells per well were cultured in triplicates in complete medium overnight. Impedance of these adherent tumor cells expressed as cell index (CI) was analyzed in 5 minutes steps over ~24 hours in a RTCA system. After reaching the linear growth phase, tumor cells were cultured with medium (spontaneous lysis) or cocultured with different indicated γδ T-cell subset lines isolated out of peripheral blood from healthy donors (open symbols, n = 3) or ovarian cancer patients (closed symbols, n = 1) at an E/T ratio of 5:1. The Vδ1 T-cell line marked with a closed rhombus is autologous to KI-OCp012 tumor cells. 12.5 IU/mL rIL-2 was added to the cultures and cells were stimulated with 1 µg/mL of bsTCE in the absence (med) or presence of the indicated concentrations of galectin-3 inhibitors TD-139. The loss of tumor cell impedance and thus a decrease of CI correlated with lysis of tumor cells. Lysis of tumor cells was measured after normalization to 1 in 3 minutes steps for additional (**A, C**) 4 or (**B, D**) 12 hours and compared to maximal lysis (100%) by Triton-X-100.

Supplemental Fig. 3

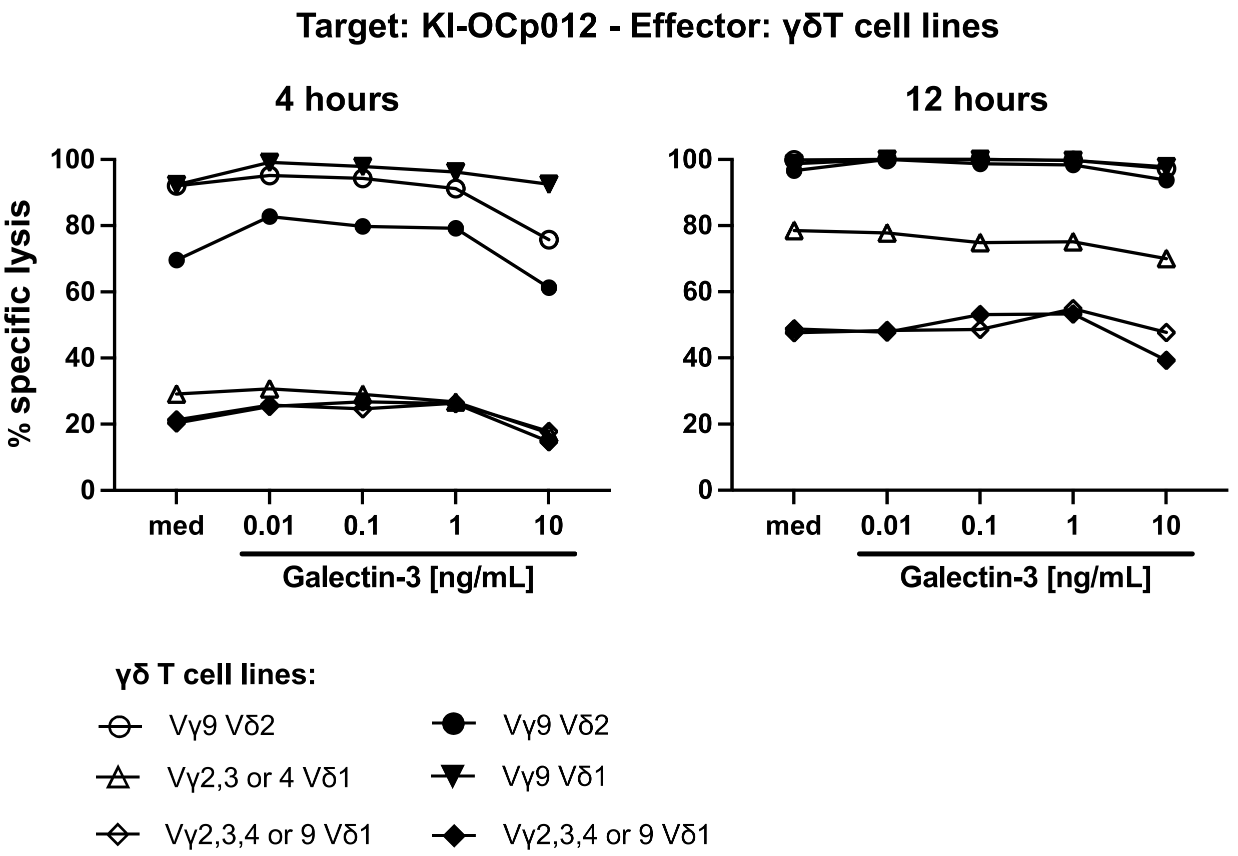

Suppl. Fig. 3 γδ T-cell cytotoxicity against ovarian cancer cells is not influenced by addition of galectin-3. A total of 10^4 indicated ovarian tumor cells per well were cultured in triplicates in complete medium overnight. Impedance of these adherent tumor cells expressed as cell index (CI) was analyzed in 5 minutes steps over ~24 hours in a RTCA system. After reaching the linear growth phase, tumor cells were cultured with medium (spontaneous lysis) or cocultured with different indicated γδ T-cell subset lines isolated out of peripheral blood from healthy donors (open symbols, n = 3) or ovarian cancer patients (closed symbols, n = 3) at an E/T ratio of 5:1. The Vδ1 T-cell line marked with a closed rhombus is autologous to KI-OCp012 tumor cells. 12.5 IU/mL rIL-2 was added to the cultures and cells were stimulated with 1 µg/mL of bispecific T-Cell Engagers in the absence (med) or presence of galectin-3 in distinct concentrations (0.01, 0.1, 1, 10 ng/mL). The loss of tumor cell impedance and thus a decrease of CI correlated with lysis of tumor cells. Lysis of tumor cells was measured after normalization to 1 in 3 minutes steps for additional 4 or 12 hours and compared to maximal lysis (100%) by Triton-X-100 at 24 hours.

Supplemental Fig. 4

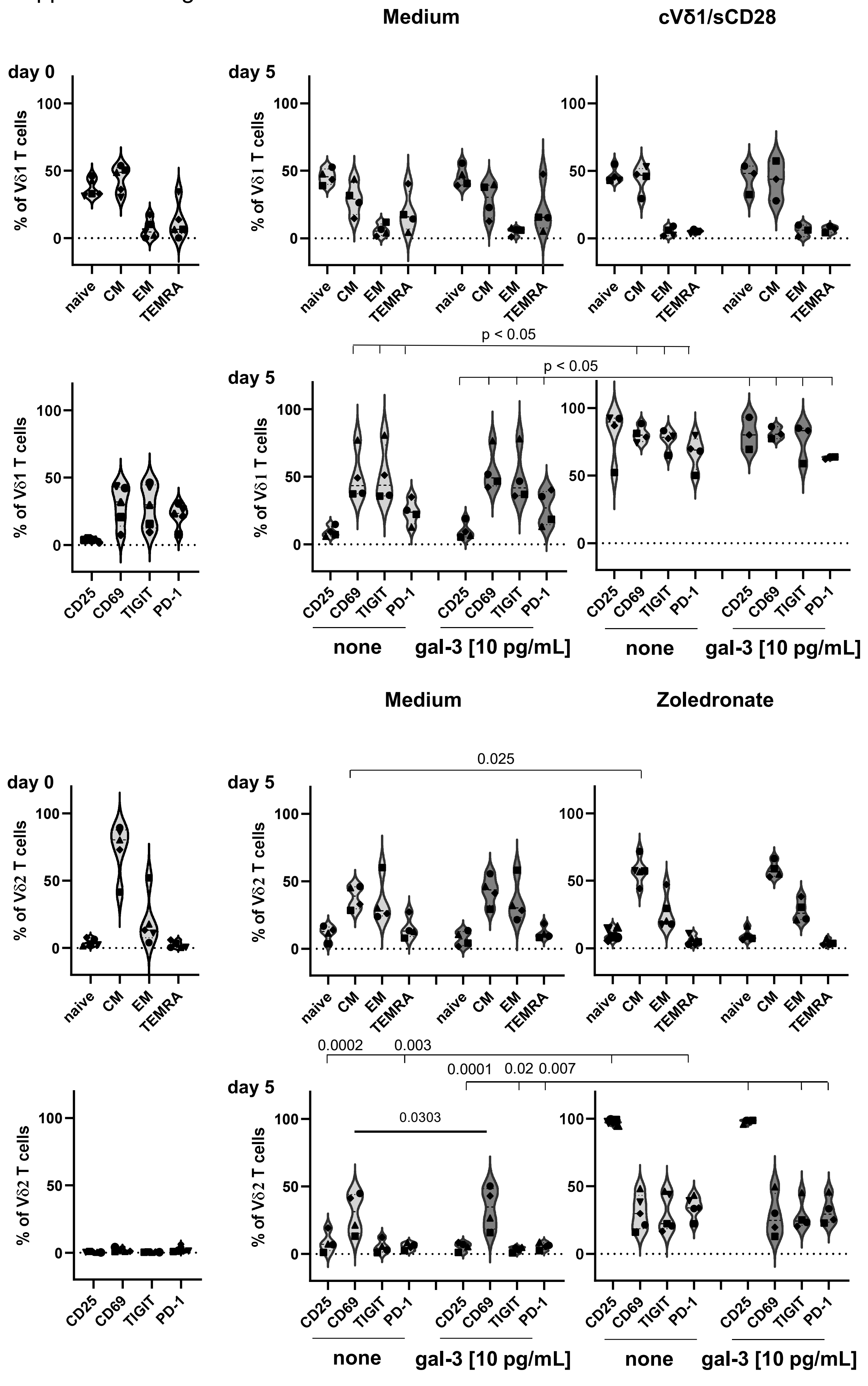

Legend of Supplemental Fig. 4

Suppl. Fig. 4 Expression of differentiation and activation on Vδ1 and Vδ2 T cells. $5x10^5$ PBMC (n = 5) were stained with anti-CD45RA and anti-CD27 mAbs to determine naïve, central and effector memory (CM and EM) or TEMRA cells of Vδ1 and Vδ2 T cells at day 0. Additionally, CD69, CD25, TIGIT and PD-1 were analyzed at day 0. Residual PBMC ($5x10^5$ cells/well) were cultured in complete medium, stimulated with 10 µg/mL coated anti-TCR Vδ1 and 1 µg/mL soluble anti-CD28 mAbs or 2.5 µM zoledronate. Medium or 10 pg/mL galectin-3 (gal-3) was added as indicated. After 5 days, cells were stained with the same mAbs as on day 0 and measured by LSR-Fortessa. A gate was set on CD45, CD3, TCRγδ and Vδ1 or Vδ2 T cells to determine naïve, CM, EM and TEMRA and the activation markers on both γδ T-cell subsets. Statistical comparison was carried out parametrically by using paired, two-tailed *t*-test. Indicated P-values are shown.

Supplemental Fig. 5

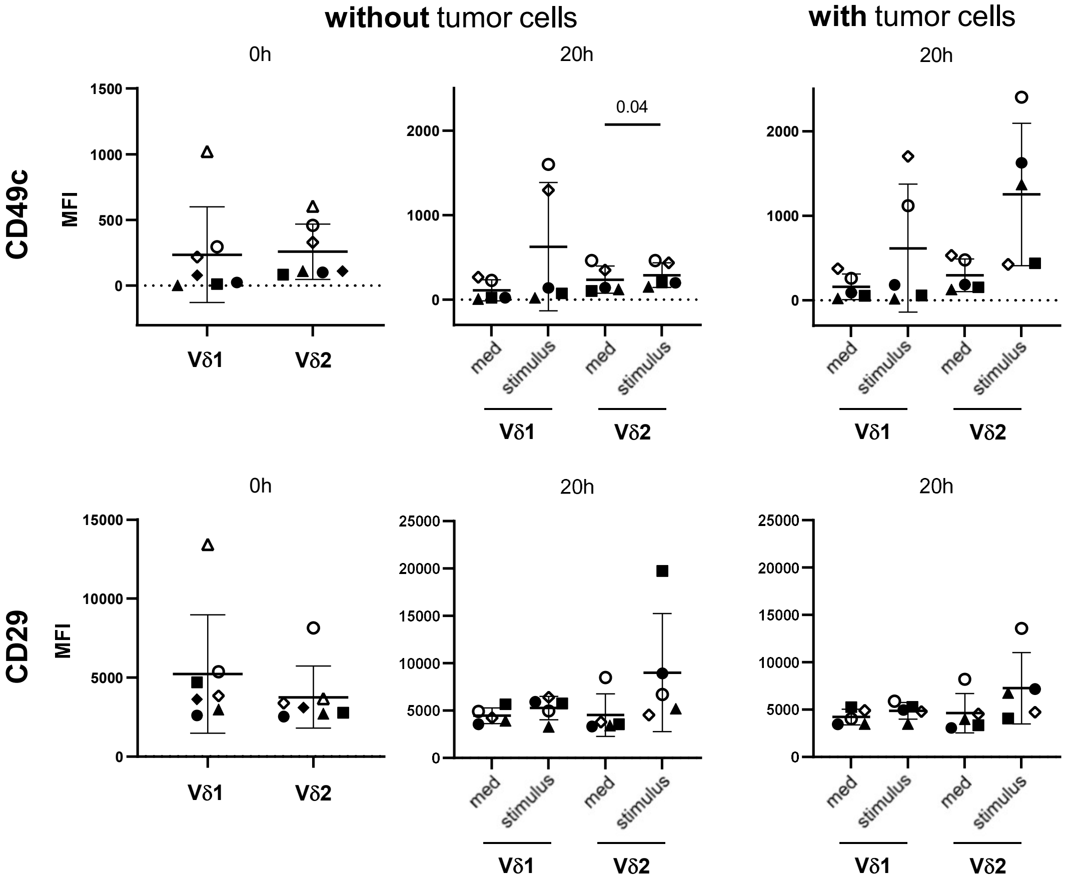

Suppl. Fig. 5 Expression of CD49c and CD29 on Vδ1 and Vδ2 T cells. $5x10^5$ PBMC (closed symbols, n = 4) and TIL (open symbols, n = 3) were stained after isolation (0 h) with anti-CD49c and anti-CD29 mAb. Residual PBMC/TIL ($5x10^5$ cells/well, without tumor cells) were cultured in complete medium, stimulated with 2.5 µM zoledronate or with coated anti-Vδ1 mAb (10 µg/mL) together with soluble anti-CD28 mAb (1 µg/mL) (stimulus). In parallel, $5x10^5$ PBMC (closed symbols) or TIL (open symbols) were co-cultured with $5x10^4$ OVCAR-3 cells (with tumor cells) in the presence of medium or bispecific T-Cell Engagers (stimulus) selectively targeting HER-2 expressing ovarian tumor cells to Vγ9Vδ2 or Vδ1 T cells. After 20 hours, cells were stained and measured by LSR-Fortessa. A gate was set on CD45, CD3, TCRγδ and Vδ1 or Vδ2 T cells to determine the CD49c and CD29 expression on both γδ T-cell subsets after 0 and 20 hours. Statistical comparison was carried out parametrically by using paired, two-tailed *t*-test or non-parametrically by using a Wilcoxon matched-pairs signed rank test. Indicated P-values are shown.
